# The impact of multiple myeloma drugs treatments on autologous stem cell transplantation in the era of new drugs

**DOI:** 10.3389/fonc.2025.1479164

**Published:** 2025-02-14

**Authors:** Xixi Wan, Tian Yu, Tao Yu, Huili Cai

**Affiliations:** ^1^ Department of Hematology, Affiliated Renhe Hospital of China Three Gorges University, Yichang, China; ^2^ College of Basic Medical Sciences, China Three Gorges University, Yichang, China; ^3^ Department of Hematology, the First Clinical Medical College of China Three Gorges University, Yichang, China

**Keywords:** multiple myeloma, chemotherapy drugs, autologous stem cell transplantation, hematopoietic stem cell mobilization, hematopoietic reconstitution

## Abstract

Autologous stem cell transplantation (ASCT) is the standard treatment recommended by the National Comprehensive Cancer Network (NCCN) for newly diagnosed multiple myeloma (MM) patients who are eligible for transplantation. This procedure follows response achieved through induction therapy. The key to the success of ASCT lies in the quantity and quality of hematopoietic stem cells collected after mobilization. Studies have shown a positive correlation between the number of hematopoietic stem cells collected and the engraftment time of absolute neutrophil count (ANC) and platelet count (PLT). However, the advent of novel therapeutic agents that have significantly improved the survival of MM patients has also impacted hematopoietic stem cell mobilization, potentially delaying hematopoietic recovery, a process referred to as hematopoietic remodeling. In this paper, we will retrospectively analyze and summarise the research progress related to the effects of previous chemotherapeutic agents on hematopoietic stem cell mobilization and hematopoietic remodeling, to further improve the prognosis and quality of survival of MM patients who are eligible for transplantation.

## 1 Introduction

Multiple myeloma (MM) is identified as a hematological cancer marked by the clonal expansion of malignant plasma cells. It constitutes about 10% of all hematological tumors and is the second most common hematologic malignancy, primarily affecting the elderly population (1). The pathogenesis of MM involves complex interactions between malignant plasma cells and the bone marrow microenvironment, including the disruption of normal hematopoiesis through cytokine dysregulation, adhesion molecule alterations, and angiogenesis. The CXCR4/SDF-1 signaling axis plays a critical role in retaining hematopoietic stem cells (HSCs) within the bone marrow, and its dysregulation in MM contributes to impaired stem cell mobilization. MM-induced osteolysis and vascular remodeling further exacerbate challenges in mobilizing HSCs for transplantation. Autologous stem cell transplantation (ASCT) is the recommended standard treatment for transplant-eligible MM patients following response through induction therapy (1). The success of ASCT depends on the effective mobilization and collection of high-quality HSCs. Hematopoietic stem cell mobilization involves the release of CD34^+^ cells from the BM into peripheral blood to achieve the minimum required yield of 2 × 10^6^/kg for a single ASCT (2). However, factors such as prior treatments with multiple myeloma drugs—including immunomodulatory drugs, proteasome inhibitors, and monoclonal antibodies (e.g., daratumumab)—and BM niche disruption can impair HSC mobilization and contribute to delays in hematopoietic reconstitution after transplantation. These delays, characterized by prolonged recovery of ANC and PLT, are often associated with stromal cell dysfunction and vascular abnormalities in the BM microenvironment. Current induction regimens for transplant-eligible MM patients have shifted from traditional chemotherapy to novel combination therapies. The VRD regimen (bortezomib, lenalidomide, dexamethasone) has emerged as the standard of care for induction therapy, offering high response rates and improved progression-free survival. Additionally, the incorporation of daratumumab into the VRD regimen (Dara-VRD) has further enhanced response depth, achieving higher rates of minimal residual disease (MRD) negativity and better overall outcomes compared to VRD alone.

The quantity and quality of hematopoietic stem cells collected after mobilization are associated with hematopoietic reconstitution post-transplantation. Key monitoring indicators for hematopoietic reconstitution include the ANC and PLT in peripheral blood. According to the World Health Organization toxicity criteria, hematopoietic reconstitution is considered delayed if either ANC > 0.5×10^9^/L or PLT > 20×10^9^/L is not achieved within 28 days (3). Studies have shown that the number of hematopoietic stem cells collected is positively correlated with the engraftment of ANC and PLT. Pathological changes in the bone marrow niche caused by MM, such as stromal cell dysfunction and vascular abnormalities, may exacerbate delays in hematopoietic reconstitution, particularly in patients exposed to prior chemotherapy regimens. Previous chemotherapy regimens may lead to delayed hematopoietic reconstitution (4).

This review aims to provide a comprehensive analysis of the effects of prior chemotherapy drugs on HSC mobilization and hematopoietic reconstitution. By summarizing current research, we explore how different chemotherapy regimens impact stem cell mobilization, with the goal of identifying strategies to optimize transplantation outcomes and improve the prognosis of MM patients.

## 2 Proteasome inhibitors

### 2.1 Bortezomib

Bortezomib, as the first-generation proteasome inhibitor, has been widely used in clinical treatment. The primary mechanism of action of bortezomib is to inhibit the normal function of the proteasome responsible for protein degradation within the cell nucleus, leading to the accumulation of proteins within the cell, which in turn triggers cellular stress and death (5). According to relevant literature, the average number of CD34^+^ stem cells collected from newly diagnosed MM patients treated with bortezomib monotherapy was 9.6×10^6^/kg (P>0.05) (6). A meta-analysis of four phase III studies (IFM2005-01, PETHEMA GEM05MENOS65, HOVON-65/GMMG-HD4, and GIMEMA MM-BO2005) involving a total of 1,572 patients was conducted to further investigate the efficacy and safety of bortezomib versus non-bortezomib regimens in transplant-eligible patients (7). In the bortezomib group (n=787), 89% of patients collected stem cells, while in the non-bortezomib group (n=785), 84% of patients collected stem cells. The average number of CD34^+^ stem cells collected was 8.33×10^6^/kg and 9.00×10^6^/kg, respectively (P>0.05). In the EVOLUTION phase II study, 170 transplant-eligible MM patients were randomized into three groups: VCD (bortezomib + dexamethasone + cyclophosphamide), VRd (bortezomib + dexamethasone + lenalidomide), and VDCR (bortezomib + dexamethasone + cyclophosphamide + lenalidomide). The average numbers of CD34^+^ stem cells collected in the three groups were 7.55×10^6^/kg(P>0.05), 7.80×10^6^/kg(P>0.05), and 6.80×10^6^/kg(P>0.05), respectively. The average ANC engraftment time was 11 days for all groups (P>0.05), and the average PLT engraftment times were 10 days (P>0.05), 10.5 days (P>0.05), and 12 days (P>0.05), respectively (8). These data suggest that bortezomib-containing induction regimens do not adversely affect stem cell mobilization and hematopoietic reconstitution.However, Moreau et al. analyzed the stem cell collection data from the IFM2005-01 trial, comparing 225 patients in the bortezomib + dexamethasone group with 216 patients in the VAD (vincristine + doxorubicin + dexamethasone) group. The average number of CD34^+^ stem cells collected in the first attempt was 6.80×10^6^/kg (P<0.001) in the bortezomib + dexamethasone group and 8.50×10^6^/kg in the VAD group. Additionally, 25% of patients in the bortezomib + dexamethasone group (P<0.05) and 13% in the VAD group required a second mobilization attempt (9). Therefore, Moreau et al. proposed that mobilization following treatment with a bortezomib-containing induction regimen tends to result in a reduced number and a slightly higher failure rate of hematopoietic stem cell collection, but the hematopoietic reconstruction recovery after transplantation is the same in both groups. These findings reveals that while bortezomib-containing regimens generally support effective mobilization and consistent engraftment, variability may arise based on regimen composition and patient-specific factors. These nuances underscore the importance of tailoring mobilization strategies to individual patient needs.

### 2.2 Ixazomib

Ixazomib is currently the first orally administered proteasome inhibitor for treating MM. It inhibits the activity of the proteasomal chymotrypsin-like β5 subunit, thereby inhibiting MM proliferation and survival with high selectivity and reversibility (10). In an early-stage trial (phase I-II) evaluating the effectiveness and safety of the IRD regimen (ixazomib + lenalidomide + dexamethasone) in the treatment of newly diagnosed multiple myeloma, 65 patients were enrolled. Twenty-nine patients underwent stem cell mobilization after completing 3-9 cycles of induction therapy, with an average collected CD34^+^ stem cell count of 11.60×10^6^/kg(P>0.05) (11). Additionally, a study on the impact of different doses of ixazomib on hematopoietic stem cell mobilization in mice also confirmed this finding. After intravenous administration of ixazomib at a dose of 8 mg/kg to mice, hematopoietic progenitor cells in peripheral blood were measured at 12, 15, and 24 hours. At 12 hours, the average number of collected hematopoietic progenitor cells was 460 CFU/ml (P>0.07), indicating a significant increase (12). These data indicate that ixazomib does not inhibit stem cell mobilization or hematopoietic reconstruction after transplantation. In another prospective phase I trial, 19 patients underwent stem cell mobilization with ixazomib + G-CSF, and the median number of collected CD34^+^ stem cells was 7.1×10^6^/kg(P>0.05). The ANC and PLT engraftment post-transplantation were 12 days (P>0.05) and 14 days (P>0.05), respectively (13). These results suggest that ixazomib does not suppress stem cell mobilization or post-transplant hematopoietic reconstruction. However, in this prospective trial, researchers believed that using ixazomib alone was not sufficient to successfully collect an adequate number of stem cells. They continuously monitored the peripheral blood CD34^+^ cell counts in two patients after ixazomib administration. In one patient, the initial CD34+ cell count post-ixazomib administration was 0.9 × 10^6^/kg, with no increase in peripheral blood CD34^+^ cells observed 10, 13, and 16 hours after administration. In another patient, the initial CD34^+^ cell count post-ixazomib was 0.2 × 10^6^/kg, and upon monitoring at the 9th hour post-administration, the CD34^+^ cell count was 1.3 × 10^6^/kg, which did not meet the target number (13). Although there are only two cases indicating a negative impact of ixazomib on hematopoietic stem cell mobilization, this also suggests that using ixazomib might adversely affect stem cell mobilization, thereby impacting the success rate of ASCT. These mixed findings emphasize the need for adjunctive agents and patient-specific assessments when incorporating ixazomib into mobilization protocols.

### 2.3 Carfilzomib

Carfilzomib is a second-generation proteasome inhibitor, which operates differently from bortezomib. Carfilzomib induces apoptosis through its specific anti-proteasomal chymotrypsin-like activity, characterized by high selectivity and irreversibility. Its efficacy and safety in patients with MM are superior to those of bortezomib (14). In a prospective study on the KRD regimen (carfilzomib + lenalidomide + dexamethasone) for newly diagnosed MM patients, 35 patients eligible for transplantation underwent stem cell mobilization after completing four cycles of KRD induction therapy. The average number of collected CD34^+^ stem cells was 6.90×10^6^/kg (P>0.05) (15). Another phase II clinical trial of the KD (carfilzomib + dexamethasone) treatment for MM patients showed an average CD34^+^ stem cell collection of 12.70×10^6^/kg(P>0.05), indicating that carfilzomib does not negatively affect hematopoietic stem cell mobilization (16). On the other hand, in a study by Susan Bal et al., the average numbers of collected CD34^+^ stem cells in the KRD and VRd groups were 9.19×10^6^/kg (P=0.02) and 11.11×10^6^/kg, respectively. Collection failures were more common in the KRD group, which might also relate to the number of cycles of lenalidomide used, but the times to ANC and PLT engraftment were comparable between the two groups (17). These findings underscores carfilzomib’s efficacy in mobilization while highlighting the influence of regimen composition on outcomes. Tailored approaches, considering patient history and drug interactions, are essential to optimize mobilization success.

## 3 Immunomodulatory drugs

In contrast to proteasome inhibitors, immunomodulatory drugs such as lenalidomide and pomalidomide present unique challenges in stem cell mobilization due to their effects on the CXCR4/SDF-1 axis. Immunomodulatory drugs, particularly lenalidomide, often impair stem cell mobilization by increasing CXCR4 expression on CD34^+^ cells, which retains them in the bone marrow niche. Pomalidomide, while effective in refractory cases, may pose similar challenges, though data are limited. These agents necessitate early intervention with plerixafor or adjusted mobilization strategies to mitigate their impact on stem cell yield.

Immunomodulatory drugs, such as lenalidomide and pomalidomide, are known to exert anti-angiogenic effects that influence the bone marrow microenvironment. These drugs modulate the expression of adhesion molecules, such as VCAM-1 and ICAM-1, which are critical for endothelial cell interactions. Furthermore, recent studies highlight their ability to disrupt signaling pathways like EGFR/HB-EGF, which are essential for endothelial cell function and new vessel formation in the bone marrow niche. By impairing angiogenesis, these agents not only inhibit tumor progression but also alter the bone marrow’s vascular architecture, potentially impacting hematopoietic stem cell mobilization. These findings underscore the dual role of immunomodulatory drugs in both anti-myeloma activity and modulation of the bone marrow microenvironment.

### 3.1 Lenalidomide

Lenalidomide, one of the most commonly used immunomodulatory drugs in multiple myeloma treatment, has been shown to significantly affect hematopoietic stem cell mobilization. Studies indicate that lenalidomide increases the expression of CXCR4 on CD34^+^ hematopoietic stem cells, which enhances their retention in the bone marrow and reduces their availability in peripheral blood. This mechanism is thought to be a major contributor to mobilization failure, particularly in patients receiving prolonged lenalidomide-based induction therapy. The International Myeloma Working Group (IMWG) guidelines recommend limiting lenalidomide exposure to no more than four cycles before hematopoietic stem cell mobilization to mitigate these risks. Additionally, the use of plerixafor in combination with granulocyte-colony stimulating factor (G-CSF) is often required to overcome lenalidomide-induced mobilization challenges and achieve adequate CD34^+^ cell yields. In a cohort of 297 patients, the median CD34^+^ cell yield was similar among those receiving more than six cycles (median 8 cycles) versus six or fewer cycles (4 cycles) of lenalidomide (p > 0.05). Mobilization was successfully achieved in over 90% of patients when plerixafor was added to G-CSF for mobilization (42). Lenalidomide maintenance therapy post-ASCT has demonstrated improved progression-free survival (PFS), with median PFS extending beyond 46 months in high-risk cytogenetic subgroups when combined with additional agents (43). Future research should prioritize optimizing induction and mobilization strategies to balance disease control and stem cell preservation.

### 3.2 Thalidomide

In the treatment of MM, the action mechanisms of immunomodulatory drugs are intricate and not yet fully elucidated (18). They primarily exert antitumor effects through influences on bone marrow angiogenesis, immunomodulatory activities, and direct cytotoxic effects on myeloma cells. Studies have confirmed that immunomodulatory drugs can alter the expression of various adhesion molecules and other proteins on myeloma cells, along with their potential anti-angiogenic effects, raising concerns about their negative impact on stem cell collection. Breitkreutz et al. analyzed data from two prospective clinical trials comparing the combination of VAD with TAD (thalidomide + doxorubicin + dexamethasone). The average numbers of collected CD34^+^ stem cells were 9.80×10^6^/kg (P=0.02) for TAD and 10.90×10^6^/kg for VAD, with the TAD group collecting fewer CD34^+^ stem cells compared to the VAD group; however, there were no differences in post-transplant engraftment kinetics between the two groups (19). Other studies have suggested that thalidomide does not affect stem cell collection. A retrospective analysis of 200 MM patients eligible for transplantation compared the efficacy of the VAD regimen with a Thalidomide + Dexamethasone regimen. The average numbers of collected CD34^+^ stem cells were 7.58×10^6^/kg (P=0.4) for the thalidomide + dexamethasone group and 10.50×106/kg for the VAD group (20). In a Phase II study of thalidomide + dexamethasone combination therapy for MM patients, stem cell collection was performed after induction therapy for 59 patients, with an average CD34^+^ stem cell count of 7.10×10^6^/kg (P>0.05), achieving the target number of stem cells required for transplantation (21). These retrospective studies and prospective studies involving the addition of thalidomide provide conflicting data regarding the impact of thalidomide on hematopoietic stem cell mobilization. Synthesizing these findings reveals that thalidomide-containing regimens may modestly reduce CD34^+^ yields compared to non-thalidomide protocols, likely due to its anti-angiogenic effects. Despite reduced yields, thalidomide regimens maintain consistent engraftment kinetics, suggesting minimal impact on hematopoietic reconstitution post-transplantation. Variability in results underscores the importance of patient-specific factors, such as disease burden and prior treatments, in determining outcomes. These observations highlight thalidomide’s nuanced impact on stem cell mobilization, emphasizing the need for individualized strategies when incorporating it into induction regimens.

### 3.3 Pomalidomide

As a third-generation immunomodulatory drug, pomalidomide has shown prominent effects in treating patients with refractory or relapsed MM, especially those who are refractory to lenalidomide and bortezomib (22). Unlike lenalidomide, pomalidomide primarily exerts its anti-inflammatory and antitumor effects by modulating cytokine levels and immune cell functions (18). In studies examining the impact of lenalidomide on hematopoietic stem cell mobilization, S Li et al. suggested that pomalidomide also increases the expression of the CXC chemokine receptor 4 on hematopoietic stem cells, thereby hypothesizing that using pomalidomide for induction could interfere with stem cell mobilization (23). However, in a prospective Phase II POMACE study, 31 MM patients underwent VPd (pomalidomide + bortezomib + dexamethasone) induction therapy. Of the 27 patients eligible for transplantation, the average number of collected CD34^+^ stem cells was 6.30 × 10^6^/kg (P>0.05). The average recovery times for ANC and PLT engraftment post-transplantation were 9 days (P>0.05) and 11 days (P>0.05), respectively, achieving the expected targets (24). Pomalidomide, as a novel immunomodulatory drug, still requires further research and exploration regarding its mechanism of action and clinical applications. The impact of pomalidomide on ASCT remains inconclusive. While these results align with expected targets, variability across patient populations warrants further investigation. Compared with other IMiDs, pomalidomide produces lower CD34^+^ yields than thalidomide in most cohorts, potentially reflecting differences in anti-angiogenic intensity. Despite lower yields, engraftment kinetics remain comparable to thalidomide and lenalidomide, highlighting its safety and efficacy for ASCT preparation. Limited data on pomalidomide’s direct mechanisms and clinical applications emphasize the need for expanded trials, particularly in patients with high-risk cytogenetics or extensive prior therapies. In conclusion, pomalidomide exhibits manageable impacts on stem cell mobilization, with clinical outcomes comparable to other IMiDs. Future studies should explore its role in tailored mobilization protocols, particularly in combination with adjunctive agents like plerixafor. While some studies suggest that lenalidomide significantly impairs stem cell mobilization by upregulating CXCR4 expression on CD34^+^ cells, others report minimal effects when combined with optimized mobilization protocols such as G-CSF and plerixafor. These inconsistencies may stem from differences in patient populations, including the extent of prior exposure to lenalidomide, variations in timing and dosing of mobilization agents, or heterogeneity in study designs. Retrospective subgroup analyses, often limited by selection bias and confounding variables, contribute to conflicting findings. To resolve these discrepancies, prospective trials directly comparing lenalidomide-containing regimens with alternative protocols are essential.

Immunomodulatory drugs, such as lenalidomide, negatively influence stem cell mobilization through their impact on adhesion molecules and bone marrow stromal interactions. Lenalidomide has been reported to upregulate CXCR4 expression on hematopoietic stem cells, enhancing their retention within the bone marrow niche and reducing their availability for peripheral mobilization. Furthermore, its anti-angiogenic effects can disrupt the vascular architecture of the bone marrow, creating a less favorable environment for stem cell egress.

## 4 Anti- CD38 monoclonal antibody

Beyond small molecule therapies, monoclonal antibodies targeting CD38 or other pathways add another layer of complexity to stem cell mobilization strategies. Monoclonal antibodies, such as daratumumab and isatuximab, significantly influence mobilization by disrupting stromal interactions and altering the bone marrow microenvironment. Although effective in treating MM, these agents often require adjunctive mobilization strategies, such as early plerixafor use, to ensure adequate stem cell collection. The variability in mobilization success observed with these agents underscores the need for tailored approaches.

### 4.1 Daratumumab

Since 2015, when CD38^+^ monoclonal antibodies were approved for the treatment of multiple myeloma patients, the therapeutic outcomes have further improved (25). Daratumumab binds specifically to the CD38^+^ antigenic epitopes on myeloma cells, exerting anti-myeloma effects through mechanisms such as antibody-dependent cellular cytotoxicity, complement-dependent cytotoxicity, antibody-dependent cellular phagocytosis, direct induction of apoptosis, and inhibition of intrinsic CD38^+^ enzymatic activity (26). However, its impact on hematopoietic stem cell mobilization and engraftment has shown variability across trials. In the phase III Cassiopeia trial, daratumumab was used in a comparative treatment of transplant-eligible MM patients using Dara-VTd (daratumumab + bortezomib + thalidomide + dexamethasone) versus VTd (bortezomib + thalidomide + dexamethasone) to study stem cell yield and transplantation outcomes (27). Among the MM patients who met the inclusion criteria, 504 in the Dara-VTd group and 490 in the VTd group underwent stem cell collection. In the Cassiopeia Phase III trial, the average numbers of collected CD34^+^ stem cells were 6.70 × 10^6^/kg (P<0.006) in the Dara-VTd group and 10.00 × 10^6^/kg in the VTd group. The use of plerixafor, a stem cell mobilizing agent, was required in 22% (P<0.001) of patients in the Dara-VTd group compared to 8% in the VTd group. Both groups showed high rates of hematopoietic reconstitution post-transplantation, with 99.8% (P>0.05) in the Dara-VTd group and 99.6% in the VTd group. This suggests that while daratumumab may complicate mobilization, it does not adversely affect engraftment kinetics. The GRIFFIN Phase II trial evaluated the efficacy and safety of adding daratumumab to the RVd (lenalidomide + bortezomib + dexamethasone) regimen along with ASCT. In this study, 207 MM patients meeting the inclusion criteria were randomized to either the Dara-RVd or RVd groups. Following four cycles of induction therapy and subsequent stem cell collection, the mean count of harvested CD34^+^ stem cells was 4.20 × 10^6^/kg (P<0.05) for Dara-RVd and 4.80 × 10^6^/kg for RVd. In the Dara-RVd group, 72% (P<0.05) of patients mobilized required the use of plerixafor compared to 55% in the RVd group. The ANC engraftment were 12 days (P>0.05) for both groups, while the average times for PLT engraftment were 12 (P>0.05) and 13 days, respectively, with comparable times for hematopoietic reconstruction (28). Both the Cassiopeia Phase III and GRIFFIN Phase II trials with daratumumab-containing quadruple therapy showed good efficacy and safety, with comparable times for hematopoietic reconstruction. The average numbers of collected CD34^+^ stem cells in both trials reached the target figures; however, they were lower than those in the control groups and required more frequent use of plerixafor. It highlights that daratumumab-containing regimens, while effective, require additional mobilization strategies to ensure adequate CD34^+^ stem cell collection.

### 4.2 Isatuximab

Isatuximab exerts its effect on CD38^+^ cells through mechanisms including Fc-dependent immune effects, immunomodulatory effects, and direct apoptotic activity. Additionally, isatuximab inhibits the extracellular enzymatic activity of CD38^+^ cells, altering calcium balance and exerting anti-myeloma effects (29). Similar to daratumumab, isatuximab-containing regimens demonstrate challenges in stem cell mobilization. In the first part of the multicenter Phase III GMMG-HD7 trial, 660 patients meeting the inclusion criteria were randomized 1:1 to the Isa-VRd (isatuximab + lenalidomide + bortezomib + dexamethasone) group or the control VRd group (30). After three cycles of induction therapy, In the Isa-VRd group, 310 patients, and in the control group, 293 patients, achieved the minimum necessary stem cell collection numbers. The average number of CD34^+^ stem cells collected was 7.71 × 10^6^/kg in the Isa-VRd group (P<0.01) compared to 9.54 × 10^6^/kg in the control group. 32% (P<0.01) of patients in the Isa-VRd group and 22% in the control group required the use of plerixafor. Compared to the control group, overall stem cell collection was impaired in the Isa-VRd group.In an investigation exploring the impaired mobilization mechanisms of CD34^+^ cells caused by anti-CD38 monoclonal antibodies, 34 MM patients eligible for transplantation received four cycles of VTd (n=14), Dara-VCd (daratumumab + bortezomib + cyclophosphamide + dexamethasone) (n=9), or Isa-KRd (isatuximab + carfilzomib + lenalidomide + dexamethasone) (n=11) before mobilization (31). The average peak concentration of CD34^+^ cells collected in the VTd group was 128.52/μL, significantly reduced to 62.75/μL (P=0.0387) and 24.90/μL (P<0.0001) in the Dara-VCd and Isa-KRd groups, respectively, compared to the VTd group. The mean number of CD34^+^ stem cells collected (×10^6^/kg) was 13.16 for VTd, and lower in the Dara-VCd and Isa-KRd groups at 10.56 (P<0.05) and 4.88 (P=0.0059), respectively. Hematopoietic engraftment time was delayed by one day in both the Dara-VCd and Isa-KRd groups compared to the VTd group (P<0.05). This study indicates that induction regimens containing CD38^+^ monoclonal antibodies affect the capacity to mobilize hematopoietic stem cells and delay hematopoiesis reconstitution time compared to other regimens. While engraftment kinetics were only slightly delayed (by one day, P<0.05), these findings underscore the impact of isatuximab on mobilization efficiency. However, in the prospective Phase II CMRG-008 trial, after four cycles of Isa-VCd (isatuximab, bortezomib, cyclophosphamide, dexamethasone) induction therapy, 65 out of 71 patients underwent hematopoietic stem cell mobilization, with an average collected CD34^+^ stem cell count of 5.30×106/kg (P>0.05). All 65 patients were successfully mobilized and their hematopoietic engraftment times met the kinetics of implantation (32). Synthesizing these studies indicates that while isatuximab-containing regimens are effective, they often necessitate plerixafor supplementation to overcome mobilization impairments.

Similarly, studies evaluating CD38 monoclonal antibodies such as daratumumab show variability in CD34^+^ yields and engraftment times. While some data indicate delayed hematopoietic reconstitution compared to non-antibody regimens, other findings suggest no significant differences. The observed inconsistencies might be attributed to differences in antibody exposure duration, patient-specific factors, or the use of adjunctive agents like plerixafor. Addressing these variations requires standardized protocols and controlled studies.

The mechanisms underlying the reduced efficiency of stem cell mobilization in patients receiving CD38+ monoclonal antibodies, such as daratumumab and isatuximab, involve alterations in the bone marrow microenvironment and adhesion molecule dynamics. Studies suggest that CD38+ antibodies can disrupt the interaction between HSCs and the bone marrow niche by modulating the CXCR4/SDF-1 signaling axis, leading to impaired mobilization. Additionally, CD38-targeted therapy has been shown to affect stromal cell function, reducing their ability to support the release of HSCs into peripheral blood. Furthermore, the direct apoptotic and immune-modulatory effects of these drugs on myeloma cells and surrounding microenvironment components may indirectly hinder stem cell mobilization efficiency.

## 5 Other chemotherapy drugs

### 5.1 Selinexor

Selinexor, a highly selective nuclear export inhibitor, binds slowly and reversibly to nuclear export protein, inhibiting the nuclear export of cargo proteins. It is primarily used in the treatment of relapsed or refractory multiple myeloma (RRMM) and is not part of current induction regimens. This correction of the abnormal localization of cargo proteins in tumor cells enables them to perform their normal functions and activities (33). In December 2021, China has authorized the combined application of selinexor and dexamethasone as a treatment for patients with relapsed and refractory MM, these patients must have previously undergone treatment with at least one proteasome inhibitor, one immunomodulatory agent, and one anti-CD38^+^ monoclonal antibody. From October 2022 to June 2023, Zhou Huixing and colleagues recruited 31 MM patients across multiple centers, and after receiving SPVD (selinexor, pomalidomide, bortezomib, and dexamethasone) induction therapy, two patients underwent ASCT post-induction without reported adverse effects on hematopoietic stem cell mobilization or delays in hematopoietic reconstitution (34). Although selinexor is in use in China, clinical experience with this drug is limited, and even more so the experience of performing ASCT after its use, making it difficult for existing studies to fully assess the specific impact of selinexor on hematopoietic stem cell mobilization and hematopoietic reconstitution.

### 5.2 Bendamustine

Bendamustine is a dual-function nitrogen mustard derivative with alkylating properties and purine analog antimetabolic effects, which acts by alkylating DNA to produce crosslinks in single or double strands, thereby disrupting synthesis and repair and exerting a toxic effect on tumor cells (35). In a Spanish multicenter Phase II study, 42 MM patients eligible for transplantation underwent 4 cycles of BVP (bendamustine, bortezomib, dexamethasone) induction therapy. Out of these, 40 MM patients underwent hematopoietic stem cell mobilization, with an average collected CD34^+^ stem cell count of 3.40×10^6^/kg (P>0.05). However, 14 patients (35%, P<0.05) failed to meet the minimum target number of CD34^+^ cells, necessitating the use of plerixafor (36). The Chinese Society of Clinical Oncology has listed chemotherapy regimens containing bendamustine as one of the high-risk factors for hematopoietic stem cell mobilization (35) and indicated that bendamustine can damage hematopoietic stem cells. It should be used with caution in patients eligible for transplantation. However, there are trials indicating that after the use of bendamustine, the collection of CD34^+^ stem cells reached target values and post-transplant hematopoietic reconstitution fully recovered. In a retrospective review of 63 MM patients who underwent hematopoietic stem cell mobilization and ASCT after pretreatment with bendamustine, researchers found that 60 of the patients successfully underwent mobilization and transplantation. The average number of collected CD34^+^ stem cells was 5.90×10^6^/kg (P>0.05), reaching the target for mobilization. The average engraftment times for ANC and PLT were 12 days (P>0.05) and 14 days (P>0.05), respectively, which conformed to the expected kinetics of engraftment (37). Another prospective Phase II research evaluated the safety and effectiveness of using bendamustine as a pretreatment regimen before the second ASCT in MM patients eligible for ASCT. After mobilization of hematopoietic stem cells, the average number of collected CD34^+^ cells was 5.00×10^6^/kg (P>0.05), with average engraftment times for ANC and PLT of 11 days (P>0.05) and 12 days (P>0.05), respectively. Complete hematopoietic reconstitution was achieved, showing good efficacy and tolerability to adverse reactions (38), providing more options and opportunities for managing MM patients. These results underscore the importance of tailored strategies when using bendamustine in transplant-eligible MM patients.

### 5.3 Cyclophosphamide

Cyclophosphamide, an alkylating agent, is occasionally used in multiple myeloma treatment under specific circumstances, such as mobilization failure or in salvage regimens. It is frequently combined with G-CSF to enhance stem cell mobilization. Cyclophosphamide exerts its effects by depleting hematopoietic progenitor cells within the bone marrow, thereby promoting the release of CD34^+^ cells into peripheral blood. Studies have shown that cyclophosphamide-based mobilization regimens can achieve adequate stem cell yields in patients with prior mobilization failure. However, its use is associated with increased risks of neutropenia, febrile episodes, and infections, necessitating close monitoring. While cyclophosphamide is not commonly part of standard induction regimens, its utility in specific clinical contexts highlights the need for tailored mobilization strategies.

## 6 Trends and comparisons across therapies

Comparing different therapeutic regimens, proteasome inhibitors such as bortezomib, ixazomib, and carfilzomib demonstrate consistent mobilization outcomes with minimal variability in CD34^+^ yields and engraftment times. Bortezomib-containing regimens are particularly reliable, although slightly reduced yields are observed when combined with agents like lenalidomide. In contrast, immunomodulatory drugs like lenalidomide and pomalidomide exhibit higher mobilization failure rates and reduced CD34^+^ yields due to their modulation of CXCR4 expression, often necessitating plerixafor. Monoclonal antibodies, including daratumumab and isatuximab, show similar trends of reduced mobilization efficiency, as evidenced by significantly lower CD34^+^ yields and increased reliance on plerixafor. Despite these challenges, engraftment times remain comparable across all regimens, underscoring the robustness of post-transplant hematopoietic recovery. These trends highlight the importance of tailored mobilization strategies, especially for therapies associated with higher mobilization inefficiencies, to optimize transplantation outcomes.

The clinical implications of stem cell yield are significant, as the amount of CD34^+^ cells collected during mobilization directly impacts transplantation success rates and outcomes. Studies have shown that higher CD34^+^ yields are associated with faster hematopoietic recovery, particularly in ANC and PLT engraftment times. For instance, patients with CD34^+^ yields exceeding 5×10^6^/kg often exhibit faster engraftment kinetics and reduced post-transplant complications compared to those with lower yields. Conversely, insufficient stem cell collection increases the risk of delayed or failed engraftment, necessitating additional mobilization attempts or prolonged recovery times.\n\n> Different therapies exhibit variable impacts on stem cell yield, influencing clinical decision-making. For example, proteasome inhibitors like bortezomib and ixazomib show consistent mobilization efficiency, minimizing the risk of inadequate stem cell collection. In contrast, therapies such as lenalidomide or CD38^+^ monoclonal antibodies require additional strategies, such as early plerixafor use, to achieve comparable yields. These interventions are particularly crucial for ensuring adequate stem cell reserves in patients undergoing ASCT. Personalizing treatment regimens based on patient-specific factors, such as prior exposure to chemotherapeutic agents or risk of mobilization failure, can further improve transplantation outcomes. For high-risk patients, implementing enhanced mobilization protocols early during induction therapy may reduce the likelihood of suboptimal yields and enhance overall transplant success rates.

The anti-angiogenic effects of immunomodulatory drugs, such as lenalidomide and pomalidomide, may be enhanced when combined with agents targeting pro-angiogenic factors, such as VEGF inhibitors. By simultaneously modulating adhesion molecules, such as VCAM-1 and ICAM-1, and suppressing pathways like VEGF/VEGFR, these combination regimens could exert additive or synergistic effects on both the bone marrow microenvironment and tumor suppression. This dual targeting approach holds promise for optimizing hematopoietic stem cell mobilization and improving transplantation outcomes. Further investigations, including preclinical and clinical studies, are warranted to evaluate the efficacy, safety, and mechanistic basis of these combination strategies. Targeting EGFR or HB-EGF represents a promising strategy to disrupt angiogenesis more directly than immunomodulatory drugs, offering a novel approach to overcome challenges in stem cell mobilization. Anti-EGFR/HB-EGF therapies may normalize the bone marrow microenvironment by reducing excess angiogenesis and vascular permeability, thereby enhancing stem cell trafficking and mobilization efficiency. Recent studies have demonstrated that HB-EGF-EGFR signaling in bone marrow endothelial cells sustains angiogenesis and tumor progression in MM (41). Blocking this pathway with agents like erlotinib or anti-HB-EGF neutralizing antibodies not only reduces angiogenic potential but also hampers MM tumor growth in preclinical models. Additionally, the integration of these therapies with immunomodulatory drugs or chemotherapy holds potential for improving long-term engraftment kinetics post-transplantation. Assessing bone marrow angiogenesis markers, such as VEGF and HB-EGF, during treatment could provide valuable insights into how these agents influence stem cell dynamics and transplantation outcomes. Further preclinical and clinical studies are warranted to evaluate the efficacy and durability of these strategies in optimizing hematopoietic stem cell mobilization and transplantation success.

Combining anti-CD38 antibodies, which modulate endothelial cells and disrupt the bone marrow microenvironment, with anti-angiogenic therapies represents a promising approach for optimizing stem cell mobilization. Anti-CD38 antibodies such as daratumumab and isatuximab can influence vascular dynamics in the bone marrow, potentially enhancing the efficacy of agents targeting angiogenic pathways like VEGF or HB-EGF inhibitors. This dual-targeting strategy may improve the efficiency of hematopoietic stem cell trafficking and collection, particularly in patients with mobilization challenges. Further research is needed to explore the mechanistic basis and clinical efficacy of such combination regimens in achieving superior transplantation outcomes.

## 7 Conclusion

The key factors affecting the success rate of ASCT are the amount and caliber of hematopoietic stem cells collected after mobilization. Studies have shown a positive correlation exists between the volume of harvested hematopoietic stem cells and the duration of engraftment for ANC and PLT (4). Moreover, the potential impact of previous chemotherapy drugs on the mobilization of hematopoietic stem cells cannot be ignored.Thus, previous chemotherapy drugs not only impact the collection of hematopoietic stem cells but may also cause delays in hematopoietic reconstitution. Lenalidomide, melphalan, and bendamustine have been definitively shown to damage hematopoietic stem cell mobilization (35). Studies indicate that lenalidomide increases the expression of the CXC chemokine receptor 4 on the surface of CD34^+^ stem cells, thereby impeding the mobilization of hematopoietic stem cells, revealing a potential mechanism for the failure of stem cell mobilization following lenalidomide induction (23). The International Myeloma Working Group recommends that when using lenalidomide, it is advisable to conduct hematopoietic stem cell collection following no more than four cycles of treatmen (39). At the same time, NCCN guidelines indicate that the use of alkylating agents can damage the stem cell reserve. For patients who may undergo ASCT, treatment regimens containing alkylating agents, especially melphalan, should be avoided (1). However, the specific impact of bendamustine on hematopoietic stem cell mobilization is currently contentious. The effects of other drugs like bortezomib, thalidomide, and newer agents such as carfilzomib, isatuximab, pomalidomide, CD38^+^ monoclonal antibodies, and selinexor, which are widely employed in patients suffering from relapsed and refractory MM, on hematopoietic stem cell mobilization are also unclear, particularly as there are few studies related to ASCT. Therefore, to ensure the success rate of transplantation, it is recommended to monitor the quantity and function of hematopoietic stem cells during the mobilization phase of induction therapy. If mobilization is inadequate, plerixafor can be used to increase the collection of stem cells, ensuring that hematopoietic function post-transplant is not compromised and reducing post-transplant adverse reactions (40). Strategies to mitigate the negative impact of monoclonal antibodies on stem cell mobilization include the early use of Plerixafor, a CXCR4 antagonist, to overcome adhesion-mediated retention of hematopoietic stem cells in the bone marrow. Studies have demonstrated that adding Plerixafor to daratumumab or isatuximab-containing regimens can significantly enhance mobilization success rates and reduce the need for repeated mobilization attempts. Additionally, optimizing mobilization regimens by combining monoclonal antibodies with G-CSF and limiting the duration of antibody exposure before mobilization can minimize adverse effects on the bone marrow microenvironment. For immunomodulatory drugs such as lenalidomide, mitigation strategies focus on shortening the duration of therapy prior to mobilization and integrating Plerixafor into mobilization protocols. For patients with a high risk of mobilization failure, such as those with extensive lenalidomide exposure, using Plerixafor earlier in combination with G-CSF has been shown to improve CD34^+^ yields. Personalized treatment plans that adjust mobilization protocols based on patient-specific factors, such as prior therapy and disease burden, can also enhance outcomes. Most drugs do not affect hematopoietic reconstitution post-transplantation; however, studies have shown that induction regimens containing CD38^+^ monoclonal antibodies may delay hematopoietic reconstitution time compared to other regimens (30). We also found that some chemotherapy drugs might negatively impact hematopoietic stem cell mobilization, although this rarely affects post-transplant hematopoietic reconstitution (See **Table 1** for details). Nevertheless, more trials are needed to fully explain the specific effects of chemotherapy drugs on the mobilization of hematopoietic stem cells and the post-transplantation outcomes concerning hematopoietic reconstitution. This will provide more valuable references for patients with MM who are eligible for transplantation when selecting treatment options.

**Table 1:** Comparison of drug classes and their impact on stem cell mobilization and transplantation outcomes in multiple myeloma.

Drug Class	Example Agents	CD34+ Yield (×10^6^/kg)	Engraftment Time (ANC/PLT)	Mobilization Failure (%)	Reliance on Plerixafor (%)
Proteasome Inhibitors	Bortezomib	8.33–9.00	10–12 days	Low	Low
	Ixazomib	11.6	12–14 days	Low	Low
	Carfilzomib	6.90–12.70	10–12 days	Moderate	Moderate
Immunomodulatory Drugs	Lenalidomide	6.30–9.00	9–11 days	High	High
	Thalidomide	7.10–9.80	10–12 days	Moderate	Moderate
	Pomalidomide	6.30–11.60	9–11 days	Moderate	Moderate
Monoclonal Antibodies	Daratumumab	6.7	12 days	High	22%
	Isatuximab	7.71	13 days	High	32%
Other Chemotherapy Drugs	Selinexor	Limited Data	Limited Data	Low	Low
	Bendamustine	3.40 – 5.90	11 – 14	Moderate	Moderate
	Cyclophosphamide	Adequate Yield	Variable	Moderate	Moderate

Ongoing research is exploring the use of novel targeted therapies and mobilization strategies to address the limitations of current approaches. For example, bispecific antibodies and CAR-T cell therapies targeting BCMA (B-cell maturation antigen) in multiple myeloma patients are being integrated into treatment regimens. While these therapies primarily focus on anti-tumor activity, their potential impact on hematopoietic stem cell mobilization remains an area of active investigation. Preliminary findings suggest that these agents may influence the bone marrow microenvironment, necessitating tailored mobilization strategies. Elranatamab, a bispecific antibody targeting BCMA and CD3, represents one such promising therapy. A study reported that elranatamab monotherapy achieved an overall response rate (ORR) of 61% in heavily pretreated relapsed or RRMM patients, demonstrating durable clinical efficacy without significantly impairing stem cell mobilization (44). Elranatamab’s mechanism of action involves recruiting T cells to target and destroy BCMA-expressing myeloma cells, which may alter the bone marrow microenvironment and potentially impact stem cell mobilization. Novel mobilization approaches are also under development, including the optimization of plerixafor dosing schedules and its combination with emerging agents such as CXCR4 antagonists beyond plerixafor. Studies are also examining the role of adjunctive therapies to improve mobilization efficiency in patients with high-risk profiles, such as those heavily pretreated with lenalidomide or monoclonal antibodies. These advancements hold promise for improving transplantation outcomes by enhancing mobilization success rates and reducing the variability associated with existing regimens. Future trials should focus on validating these novel strategies in prospective, controlled settings to establish their safety and efficacy.

The current evidence on the impact of various therapies on hematopoietic stem cell mobilization and transplantation outcomes has significant limitations. Much of the available data is derived from subgroup analyses within larger trials, rather than head-to-head prospective studies, introducing potential biases and reducing the generalizability of findings. Additionally, heterogeneity in patient characteristics, treatment protocols, and study designs complicates direct comparisons between regimens. Sample sizes in many studies are relatively small, limiting statistical power and the ability to draw robust conclusions. Future research should prioritize well-designed, prospective, and comparative trials to evaluate different induction and mobilization strategies in homogenous patient cohorts. Expanding the scope to include diverse patient populations and real-world data will also enhance the applicability of findings, ultimately guiding optimized treatment strategies for transplant-eligible MM patients.
